# Race and Ethnicity and Diagnostic Testing for Common Conditions in the Acute Care Setting

**DOI:** 10.1001/jamanetworkopen.2024.30306

**Published:** 2024-08-27

**Authors:** Michael I. Ellenbogen, P. Logan Weygandt, David E. Newman-Toker, Andrew Anderson, Nayoung Rim, Daniel J. Brotman

**Affiliations:** 1 Department of Medicine, Johns Hopkins School of Medicine, Baltimore, Maryland; 2 Department of Emergency Medicine, Johns Hopkins School of Medicine, Baltimore, Maryland; 3 Armstrong Institute Center for Diagnostic Excellence, Johns Hopkins School of Medicine, Baltimore, Maryland; 4 Department of Neurology, Johns Hopkins School of Medicine, Baltimore, Maryland; 5 Department of Health Policy and Management, Johns Hopkins Bloomberg School of Public Health, Baltimore, Maryland; 6 Department of Economics, US Naval Academy, Annapolis, Maryland

## Abstract

**Importance:**

Overuse of diagnostic testing is pervasive, but the extent to which it varies by race and ethnicity in the acute care setting is poorly understood.

**Objective:**

To use a previously validated diagnostic intensity index to evaluate differences in diagnostic testing rates by race and ethnicity in the acute care setting, which may serve as a surrogate for diagnostic test overuse.

**Design, Setting, and Participants:**

This was a cross-sectional study of emergency department (ED) discharges, hospital observation stays, and hospital admissions using administrative claims among EDs and acute care hospitals in Kentucky, Maryland, North Carolina, and New Jersey, from 2016 through 2018. The diagnostic intensity index pairs nonspecific principal discharge diagnoses (nausea and vomiting, abdominal pain, chest pain, and syncope) with related diagnostic tests to estimate rates of nondiagnostic testing. Adults with an acute care encounter with a principal discharge diagnosis of interest were included. Data were analyzed from January to February 2024.

**Exposure:**

Race and ethnicity (Asian, Black, Hispanic, White, other [including American Indian, multiracial, and multiethnic], and missing).

**Main Outcomes and Measures:**

Receipt of a diagnostic test. Generalized linear models with a hospital-specific indicator variable were estimated to calculate the adjusted odds ratio of receiving a test related to the principal discharge diagnosis by race and ethnicity, controlling for primary payer and zip code income quartile.

**Results:**

Of 3 683 055 encounters (1 055 575 encounters [28.7%] for Black, 300 333 encounters [8.2%] for Hispanic, and 2 140 335 encounters [58.1%] for White patients; mean [SD] age of patients with encounters, 47.3 [18.8] years; 2 233 024 encounters among females [60.6%]), most (2 969 974 encounters [80.6%]) were ED discharges. Black compared with White patients discharged from the ED with a diagnosis of interest had an adjusted odds ratio of 0.74 (95% CI, 0.72-0.75) of having related diagnostic testing. No other racial or ethnic disparities of a similar magnitude were observed in any acute care settings.

**Conclusions and Relevance:**

In this study, White patients discharged from the ED with a nonspecific diagnosis of interest were significantly more likely than Black patients to receive related diagnostic testing. The extent to which this represents diagnostic test overuse in White patients vs undertesting and missed diagnoses in Black patients deserves further study.

## Key Points

**Question:**

For common conditions in the acute care setting, are there differences in diagnostic testing across racial and ethnic groups?

**Findings:**

In this cross-sectional study of 3 683 055 encounters among patients with a principal discharge diagnosis from the emergency department of nausea or vomiting, abdominal pain, chest pain, or syncope, Black patients had lower odds of having related diagnostic testing compared with White patients.

**Meaning:**

These findings suggest that White patients discharged from the emergency department with these common diagnoses may receive higher levels of diagnostic test overuse than Black patients and that Black patients may be at increased risk for missed diagnoses.

## Teaser

This cross-sectional study investigates the association of race and ethnicity with rates of diagnostic testing among patients in acute care.

## Introduction

Racial and ethnic disparities in health care provision can drive inequity in outcomes. Previous work evaluating the association of race and ethnicity with low-value care has focused on the Medicare fee-for-service population.^1,2^ These studies have used measures from the Milliman MedInsight Health Waste Calculator^3^ and the Choosing Wisely campaign,^4^ focusing on outpatient screening, diagnostics, and therapeutics.

The degree to which low-value care varies by race and ethnicity in the acute care setting and in younger populations is not well understood. Prior research has focused on racial and ethnic differences in the rate of imaging in the emergency department (ED) and found that Black patients typically received less imaging than White patients.^5,6^ These studies have focused on broad rates of imaging in the ED or on patients who present to the ED with a specific complaint.

A previously validated hospital-level diagnostic intensity index (DII) pairs nonspecific principal discharge diagnoses with diagnostic tests to infer hospital-level rates of nondiagnostic testing and uses this to stratify hospitals by level of testing overuse.^7^ Prior work showing no association of hospital DII value with disease-specific 30-day readmissions and mortality^8^ supports the notion that the DII may be a useful proxy for test overuse. We applied this DII to patients in the ED and hospital setting to understand the association between race and ethnicity and diagnostic test overuse.

## Methods

This cross-sectional study is reported following the Strengthening the Reporting of Observational Studies in Epidemiology (STROBE) reporting guideline. Institutional review board approval was not obtained based on a waiver received from Johns Hopkins School of Medicine for limited datasets. In accordance with this, consent of study participants was not required.

### Application of Previously Developed Hospital-Level DII to the Patient Level

We used a DII that pairs nonspecific principal discharge diagnoses with diagnostic testing.^7^ Coding guidelines require that the principal discharge diagnosis for an ED visit or hospitalization can be in the “symptoms, signs, or ill-defined condition” category only if no more specific diagnosis is made.^9^ Thus, it is expected that a nonspecific principal discharge diagnosis paired with a diagnostic test implies a nondiagnostic or negative test result. Hospitals are incentivized to follow this coding guidance to optimize reimbursement and risk adjustment.

Metrics ultimately included in the DII after derivation-validation were nausea/vomiting and abdomen/pelvis computed tomography (CT) or upper endoscopy, abdominal pain and abdomen/pelvis CT or upper endoscopy, chest pain and chest CT or stress test, and syncope and brain CT or stress test.^7^ The DII was externally validated with Dartmouth Atlas hospital service area use measures and Medicare risk-adjusted county-level cost measures.^7^

To apply the DII to the patient level, we included all ED visits and hospitalizations with a principal discharge diagnosis of interest. These were identified using *International Statistical Classification of Diseases and Related Health Problems, Tenth Revision *(*ICD-10*) diagnosis codes (eTable 1 in Supplement 1). Whether patients underwent the associated diagnostic testing was determined using *ICD-10* procedure, Current Procedural Terminology, and revenue codes.

### Diagnostic Terminology

The term *diagnostic intensity* describes rates of diagnostic test use. Low-value care is defined as “a health service for which the harms or costs outweigh the benefits.”^10^ This could take the form of screening, diagnostics, or procedures. Our DII focuses on diagnostics and uses the yield of information from testing (more specifically, rates of nondiagnostic testing) to infer potential test overuse (synonymous with *diagnostic* low-value care). Based on a large body of evidence suggesting pervasive diagnostic test overuse,^11,12,13,14,15,16^ we suspect that for groups of patients (whether clustered by hospital, race and ethnicity, or other characteristics), a high level of diagnostic intensity is associated with higher levels of test overuse. Additionally, groups with high rates of diagnostic testing with a resulting low yield of information (ie, high rates of nondiagnostic testing) are also likely receiving higher levels of diagnostic test overuse.^17,18,19^ However, relatively low rates of diagnostic testing with or without a higher yield of information may be associated with more missed diagnoses.

### Data Source, Inclusion Criteria, and Study Design

Administrative data from the State ED Databases (SEDD) and State Inpatient Databases (SID) of the Healthcare Cost and Utilization Project (HCUP) were obtained.^20^ We used the SEDD to identify ED visits not leading to hospitalization and observation stays not leading to inpatient admissions. We used the SID to identify inpatient hospitalizations. Our data included all ED visits and hospitalizations from 2016 through 2018 in Kentucky, Maryland, New Jersey, and North Carolina. These states were chosen based on cost and availability of relevant data elements. We included all encounters for patients aged 18 years or older with a principal discharge diagnosis of interest. Encounters at pediatric, psychiatric, long-term acute care, and specialty hospitals were excluded.

### Race and Ethnicity Categories

HCUP data on race and ethnicity for the SID and SEDD come from state partner organizations that use varying levels of granularity. HCUP created a uniform variable that divides race and ethnicity into the following categories: Asian, Black, Hispanic, American Indian, White, and other, as well as a missing category. The other category includes patients with more than 1 listed racial or ethnic category and those listed as other. For this analysis, we combined American Indian patients with other owing to the small number of American Indian observations. The HCUP uniform race variable combines patients categorized as Hispanic White, Hispanic Black, Hispanic other, and Hispanic unspecified in a separate ethnicity variable into the Hispanic category.

### Descriptive Analysis

Encounters were analyzed by care setting (ED visits not leading to hospitalization, observation stays not leading to admission, and inpatient admissions). These groups were characterized by mean (SD) age and percentage female. The racial and ethnic composition across care settings, primary payer (Medicare, Medicaid, commercial, self-pay, other, and missing), and diagnosis of interest was described. Additionally, the mean Elixhauser score^21^ was calculated for each racial and ethnic group in each setting. We also reported the proportion of encounters with each principal diagnosis of interest with associated diagnostic testing, as well as the proportion of patients with any principal diagnosis of interest with associated testing in each setting.

### Statistical Analysis

#### Main Analysis

For each setting, we estimated univariate and multivariable generalized linear models at the encounter level with a hospital-specific indicator variable using a logit link function. For each model, a binary indicator for performance of the relevant diagnostic test was the outcome, and the factor of interest was race and ethnicity. Additional factors in the multivariable model were primary payer and quartiled median income of patient zip code of residence, which may be strongly associated with race and ethnicity. We created a missing category for both race and ethnicity and primary payer. Encounters for which zip code income quartile was missing were excluded from the analysis. Analyses were performed using R statistical software version 4.4.1 (R Project for Statistical Computing). Data were analyzed from January to February 2024. Results were considered significant at *P* < .05 in 2-sided tests.

#### Subanalyses and Sensitivity Analyses

We performed subanalyses by zip code income quartile (with primary payer as a covariate), for Medicaid patients only (with zip code income quartile as a covariate), for self-pay patients only (with zip code income quartile as a covariate), for patients who were unhoused (univariate analysis), and by age (<65 years and ≥65 years, with primary payer and zip code income quartile as covariates, although restricting to Medicare patients for those aged ≥65 years). In Medicaid and self-pay subanalyses, we included only patients younger than 65 years. Finally, we conducted a subanalysis of inpatient hospitalizations that did not begin in the ED.

We conducted a sensitivity analysis adding patient sex, age, and Elixhauser score as additional covariates. We also conducted a sensitivity analysis excluding the hospital-specific indicator variable. Finally, we conducted a sensitivity analysis using the ED chief complaint for ED visits and observation stays and the admitting diagnosis for inpatient hospitalizations rather than the principal discharge diagnosis. The admitting diagnosis variable was not present in Maryland SID data, so Maryland encounters were excluded from this analysis.

## Results

### Descriptive Statistics

Our sample included 3 683 055 encounters (1 055 575 encounters [28.7%] for Black, 300 333 encounters [8.2%] for Hispanic, and 2 140 335 encounters [58.1%] for White patients; mean [SD] age of patients with encounters, 47.3 [18.8] years; 2 233 024 encounters among females [60.6%]) (Table 1). Most encounters (2 969 974 encounters [80.6%]) were for patients discharged from the ED. Black patients were hospitalized at a lower rate than White patients (186 131 encounters [17.6%] vs 446 333 encounters [20.9%]). Black and White patients had a mean (SD) age of 44.9 (17.3) years and 49.6 (19.0) years, respectively.

**Table 1.** Demographics, Care Settings, and Diagnoses by Race and Ethnicity

Race and ethnicity	Encounters, No. (%)				Age, mean (SD), y	% Female	Diagnoses, No. (%)			
	Total	ED	Observation	Inpatient			Nausea or vomiting	Abdominal pain	Chest pain	Syncope
Asian	45 414 (1.2)	34 485 (1.2)	9580 (1.6)	1349 (1.4)	48.9 (18.4)	27 078 (59.6)	4047 (1.0)	15 400 (1.2)	20 380 (1.3)	5587 (1.6)
Black	1 055 575 (28.7)	869 444 (29.3)	162 059 (26.2)	24 072 (25.6)	44.9 (17.3)	663 133 (62.8)	119 936 (30.9)	373 604 (27.9)	477 803 (29.7)	84 232 (24.2)
Hispanic	300 333 (8.2)	255 963 (8.6)	38 393 (6.2)	5977 (6.4)	41.9 (16.5)	184 422 (61.4)	26 741 (6.9)	137 640 (10.3)	116 771 (7.3)	19 181 (5.5)
White	2 140 335 (58.1)	1 694 002 (57.0)	386 833 (62.50)	59 500 (63.2)	49.6 (19.0)	1 275 334 (59.6)	223 135 (57.5)	755 045 (56.5)	935 709 (58.1)	226 446 (65.0)
Other^a^	117 773 (3.2)	96 739 (3.3)	18 375 (3.0)	2659 (2.8)	43.6 (17.4)	68 960 (58.6)	11 985 (3.1)	46 663 (3.5)	48 852 (3.0)	10 273 (3.0)
Missing	23 625 (0.6)	19 341 (0.7)	3733 (0.6)	551 (0.6)	44.5 (18.2)	14 097 (59.7)	2350 (0.6)	8855 (0.7)	9800 (0.6)	2620 (0.8)
Total	3 683 055 (100)	2 969 974 (100.0)	618 973 (100.0)	94 108 (100.0)	47.3 (18.8)	2 233 024 (60.6)	388 194 (100.0)	1 337 207 (100.0)	1 609 315 (100.0)	348 339 (100.0)

Abbreviation: ED, emergency department.

^a^ Other race and ethnicity is defined as American Indian, more than 1 reported racial or ethnic category, and patients who selected other as their race or ethnicity.

The most common diagnosis group for the cohort was chest pain, followed by abdominal pain (Table 1). For each racial and ethnic group, commercial payer was the most common primary payer (eTable 2 in Supplement 1). Black and White patients discharged from the ED both had a mean (SD) Elixhauser score of 0.9 (1.2) (eTable 3 in Supplement 1).

The lowest rate of testing was 55 633 of 360 228 encounters (15.4%) for patients in the ED with nausea and vomiting, and the highest rate of testing was 11 691 of 15 005 encounters (77.9%) for inpatients with abdominal pain (Table 2). The overall rates of testing were 960 038 of 2 969 974 encounters (32.3%), 387 121 of 618 973 encounters (62.5%), and 61 865 of 94 108 encounters (65.7%) for ED discharges, observation stays, and inpatient hospitalizations, respectively.

**Table 2.** Rates of Diagnostic Testing by Care Setting

Metric	Setting, No. of tests/total cases (%)		
	ED discharges	Observation stays	Inpatient admissions
Nausea/vomiting and abdominal/pelvis CT or upper endoscopy	55 633/360 228 (15.4)	9084/20 031 (45.3)	4244/7935 (53.5)
Abdominal pain and abdominal/pelvis CT or upper endoscopy	635 877/1 261 977 (50.4)	44 098/60 225 (73.2)	11 691/15 005 (77.9)
Chest pain and chest CT or stress test	177 992/1 124 410 (15.8)	258 853/439 813 (58.9)	25 853/45 092 (57.3)
Syncope and brain CT or stress test	90 536/223 359 (40.5)	75 086/98 904 (75.9)	20 077/26 076 (77.0)
Any diagnosis of interest with associated testing	960 038/2 969 974 (32.3)	387 121/618 973 (62.5)	61 865/94 108 (65.7)

Abbreviations: CT, computed tomography; ED, emergency department.

### Main Statistical Analysis

Black patients compared with White patients discharged from the ED with a diagnosis of interest had an adjusted odds ratio (aOR) of 0.74 (95% CI, 0.72-0.75) of having related testing (Table 3; eTable 4 in Supplement 1). For observation stays and inpatient hospitalizations, aORs were 0.92 (95% CI, 0.90-0.94) and 1.00 (95% CI, 0.95-1.05), respectively.

**Table 3.** Racial and Ethnic Differences in Receiving Diagnostic Testing by Acute Care Setting

Race and ethnicity	Unvariate analysis		Multivariable analysis	
	aOR (95% CI)^a^	*P* value	aOR (95% CI)^a^	*P* value
**ED**				
Asian	0.90 (0.87-0.92)	<.001	0.91 (0.89-0.94)	<.001
Black	0.71 (0.69-0.72)	<.001	0.74 (0.72-0.75)	<.001
Hispanic	0.92 (0.90-0.94)	<.001	1.00 (0.98-1.02)	.93
Other^b^	0.88 (0.83-0.92)	<.001	0.93 (0.89-0.97)	.002
Missing	0.76 (0.72-0.81)	<.001	0.81 (0.76-0.85)	<.001
**Observation**				
Asian	1.09 (1.04-1.15)	<.001	1.08 (1.03-1.13)	.002
Black	0.92 (0.90-0.94)	<.001	0.92 (0.90-0.94)	<.001
Hispanic	1.05 (1.01-1.09)	.02	1.03 (1.00-1.07)	.06
Other^b^	1.03 (0.99-1.08)	.09	1.02 (0.99-1.06)	.22
Missing	1.12 (1.04-1.21)	.002	1.11 (1.03-1.19)	.009
**Inpatient**				
Asian	1.12 (1.00-1.26)	.05	1.13 (1.01-1.27)	.04
Black	0.98 (0.93-1.03)	.37	1.00 (0.95-1.05)	.90
Hispanic	1.06 (0.99-1.14)	.10	1.09 (1.02-1.17)	.02
Other^b^	0.99 (0.91-1.09)	.91	1.01 (0.92-1.10)	.85
Missing	0.83 (0.67-1.03)	.09	0.83 (0.67-1.03)	.09

Abbreviations: aOR, adjusted odds ratio; ED, emergency department.

^a^ This was a generalized linear model with a hospital-specific indicator variable, adjusting for primary payer and zip code income quartile. The aOR represents the odds ratio of a patient from a specific racial or ethnic group having a diagnostic test compared with White patients (the reference group for all comparisons).

^b^ Other race and ethnicity is defined as American Indian, more than 1 reported racial or ethnic category, and patients who selected other as their race or ethnicity.

### Subanalyses

The finding of Black patients discharged from the ED receiving lower rates of testing was consistent in the subanalysis across zip code income quartiles (eTables 5-7 in Supplement 1). In the Medicaid subanalysis, Black patients in the ED were still significantly less likely to undergo testing, with an aOR of 0.72 (95% CI, 0.70-.073) (eTable 8 in Supplement 1).

Similar trends were observed in subanalyses by self-pay status (eTable 9 in Supplement 1), unhoused status (eTable 10 in Supplement 1), and age (Table 4). Findings were also similar in the subanalysis of inpatient hospitalizations that did not begin in the ED, although Hispanic patients had a higher rate of testing (eTable 11 in Supplement 1).

**Table 4.** Age Subanalysis of Racial and Ethnic Differences in Receiving Diagnostic Testing by Acute Care Setting

Setting	Age <65 y		Age≥65 y	
	aOR (95% CI)^a^	*P* value	aOR (95% CI)^a^	*P* value
ED				
Asian	0.88 (0.85-0.91)	<.001	1.03 (0.96-1.10)	.37
Black	0.72 (0.71-0.73)	<.001	0.93 (0.90-0.96)	<.001
Hispanic	0.99 (0.97-1.01)	.35	1.10 (1.04-1.16)	<.001
Other^b^	0.91 (0.87-0.95)	<.001	1.14 (1.06-1.22)	<.001
Missing	0.79 (0.74-0.83)	<.001	0.95 (0.85-1.06)	.36
Observation				
Asian	1.02 (0.96-1.08)	.62	1.13 (1.04-1.23)	.003
Black	0.93 (0.90-0.95)	<.001	0.94 (0.90-0.97)	<.001
Hispanic	1.04 (1.00-1.08)	.09	1.04 (0.96-1.13)	.32
Other^b^	1.01 (0.96-1.06)	.69	1.06 (0.99-1.14)	.09
Missing	1.11 (1.00-1.23)	.05	1.13 (0.99-1.28)	.06
Inpatient				
Asian	1.16 (0.98-1.37)	.09	1.04 (0.87-1.24)	.66
Black	1.01 (0.95-1.07)	.84	1.04 (0.96-1.12)	.35
Hispanic	1.13 (1.04-1.24)	.005	1.01 (0.88-1.16)	.91
Other^b^	1.02 (0.90-1.15)	.80	0.95 (0.81-1.11)	.51
Missing	0.79 (0.61-1.01)	.07	0.88 (0.61-1.27)	.49

Abbreviations: aOR, adjusted odds ratio; ED, emergency department discharges.

^a^ This shows the subanalysis by age using a generalized linear model with a hospital-specific indicator variable. For the younger than 65 years group, we adjusted for primary payer and zip code income quartile. For the age 65 and older group, we adjusted for zip code income quartile and restricted to Medicare patients. The aOR represents the OR of a patient from a specific racial or ethnic group having a diagnostic test compared with White patients (the reference group for all comparisons).

^b^ Other is defined as American Indian, more than 1 reported racial or ethnic category, and patients who selected other as their race.

### Sensitivity Analyses

The sensitivity analysis adding sex, age, and Elixhauser score covariates had similar findings to the main model (eTable 12 in Supplement 1). The sensitivity analysis without the hospital-specific indicator variable also had similar findings but with a mildly increased magnitude (eTable 13 in Supplement 1). In the sensitivity analysis using reason for presentation instead of principal diagnosis, Black patients discharged from the ED received less diagnostic testing than White patients, with a similar magnitude to the main analysis (eTables 14 and 15 in Supplement 1).

## Discussion

In this cross-sectional study, Black patients discharged from the ED with a principal discharge diagnosis of interest were less likely than White patients to have associated diagnostic testing. This finding persisted in subanalyses. This disparity was also observed in observation stays, although the magnitude was smaller. No Black-White disparity was observed for inpatients. Disparities of clinically meaningful magnitudes for other racial and ethnic groups were not consistently observed in any acute care settings.

This Black-White disparity is consistent with prior work. Research on diagnostic imaging rates using the National Hospital Ambulatory Medical Care Survey (NHAMCS) between 2005 and 2016 found that Black patients were 16% less likely to receive any imaging in the ED.^22^ A systematic review of the association between race and ED imaging found that Black patients presenting to the ED with abdominal pain or trauma were generally less likely to receive diagnostic imaging.^6^ A single-center study using 2016 data^23^ and a study using NHAMCS data from 2005 through 2014^24^ found that Black patients with abdominal pain had an aOR of 0.58 of undergoing a CT scan in the ED compared with White patients. A 2001 study^25^ of 2 EDs found that Black patients presenting with chest pain were more likely to undergo echocardiogram but less likely to undergo cardiac catheterization than White patients. An analysis of NHAMCS data from 1995 through 2000 found that Black patients presenting to the ED with chest pain were less likely to receive a chest x-ray than patients of other races.^26^

In addition to including a large number of hospitals and a range of discharge diagnoses, our analysis builds on previous work in another important way: looking at imaging paired with nonspecific principal discharge diagnoses rather than overall rates of imaging or rates of imaging for a specific chief complaint allowed us to infer rates of nondiagnostic testing. Prior work suggests that a low yield of useful information from diagnostic imaging may be a proxy for test overuse.^17,18,19,27^ White patients discharged from the ED had higher rates of testing paired with nonspecific principal diagnoses than Black patients and thus higher rates of nondiagnostic testing for conditions of interest. This suggests that White patients may receive more test overuse but also raises the concern that undertesting among Black patients may lead to more missed diagnoses. To our knowledge, racial and ethnic differences in the information yield of diagnostic testing have not been previously studied.

Prior research on racial and ethnic differences in provision of low-value care is mixed and has focused on older patients and the outpatient setting. A systematic review^28^ studying the association between race and ethnicity and overuse examined 59 studies, including 74 outcomes. For 43% of outcomes, White patients received more low-value care. For the remaining 57% of outcomes, there was no racial and ethnic difference (45%) or members of minority racial and ethnic groups received more low-value care (12%). However, some studies contradict this pattern. For example, 2 studies of Medicare patients focused on outpatients. A 2017 study^1^ found that receipt of low-value services was higher among Black patients than White patients for 5 of 11 services. A 2023 study^2^ found that Black compared with White patients were more likely to receive low-value acute diagnostic testing and less likely to receive low-value screening tests and treatment.

Drivers of disparities we observed are unclear. However, there is strong evidence of racial bias in the ED triage process, with Black patients receiving lower-triage acuity than clinically similar White patients.^22,29,30,31^ Even among high-acuity patients, Black patients may be more likely to be triaged to a fast-track system than White patients.^32^ Triage disparities could, in turn, bias the clinician who assumes care after triage but could also impact where the patient is placed in the ED, tests ordered, and type of clinician a patient sees. Another potential explanation is that communication difficulty between physicians who are predominantly White^33^ and Black patients leads to these patients’ symptoms being dismissed more often. The extent to which our findings are associated with differences in access to primary care is unclear. It may be anticipated, for instance, that patients using the ED for medical issues that might be addressed in a primary care setting (such as upper respiratory infections or chronic back pain) may receive different intensities of diagnostic testing than the broader ED population. However, our focus on acute and potentially serious symptom presentations that often require time-sensitive evaluation may mitigate this issue.

Finally, implicit bias may contribute to these findings. A 2015 systematic review^34^ found evidence that most clinicians have more positive attitudes toward White patients and negative attitudes toward patients of other races and ethnicities. Cognitive psychology research suggests that implicit bias is more likely to impact type 1 decisions (faster, using fewer cognitive resources) than type 2 decisions (slower, using more cognitive resources).^35^ The fact that ED clinicians face greater time pressure and thus make more type 1 decisions than those taking care of hospitalized patients may be a reason this disparity was observed for patients discharged from the ED but not those who were hospitalized.

### Limitations

Our study has limitations. We focused on racial and ethnic differences in test overuse but did not study test underuse, limiting our ability to evaluate variation in missed diagnoses across races and ethnicities. Without longitudinal data, we were unable to determine whether our findings showing less test overuse for patients discharged from the ED were accompanied by test underuse and more missed diagnoses. We suspect that Black patients in the ED receive less test overuse but also are more likely to have a missed diagnosis, and the literature supports both hypotheses. A 2017 analysis of Medicare Advantage and commercially insured patients measured a nearly 60% increase in CT imaging during ED visits from 2005 to 2013.^36^ This trend has been associated with empirical evidence of overuse of ED imaging for the symptoms we evaluated,^18,37,38,39^ as well as survey studies of ED physicians acknowledging overuse of CT imaging.^40,41^ However, a systematic review of factors associated with overuse (rather than simply use) of imaging in the ED noted only 1 study on the association between race and overuse of ED imaging.^42^ It found that White patients with trauma were more likely than Black patients with trauma to undergo duplicate CT imaging.^43^

Black patients presenting to the ED with gastrointestinal symptoms had higher rates of missed appendicitis than White patients.^44^ Black patients presenting to the ED with chest pain were more likely to experience a missed myocardial infarction than White patients.^45,46^ An analysis of NHAMCS data from 2005 through 2016 found that Black patients were 10% less likely than White patients to be admitted and 1.3 times more likely to die in the ED or hospital.^22^

In addition to our inability to determine rates of missed diagnoses, there are other limitations. First, we were unable to fully adjust for patient education and income level. More educated or affluent patients may prefer more testing. Zip code income quartile is an imperfect measure of socioeconomic status. Second, although our model adjusted for clustering at the hospital level, there is potential for confounding from variation in coding intensity across hospitals. More intensive coding could lead to fewer nonspecific principal discharge diagnoses in general, and to the extent that a hospital’s coding intensity is associated with the racial and ethnic demographics of its patient population, this could impact results. Third, given prior research showing bias in ED triage,^22,29,30,31,32^ our lack of data on ED triage is a limitation.

## Conclusions

In this cross-sectional study, we applied a hospital-level DII to patients with ED visits or hospitalizations to determine if rates of nondiagnostic testing varied by race and ethnicity. White patients discharged from the ED had higher rates of nondiagnostic testing, suggesting higher levels of testing overuse. Although Black patients were subjected to less test overuse, this may have come at a risk of undertesting and missed diagnoses. Future research should attempt to better understand the cause of diagnostic racial and ethnic disparities and evaluate whether they are associated with missed diagnoses.

## Notes

Supplement 1. **eTable 1.** Diagnostic and Procedural Codes for Metrics **eTable 2.** Payer Mix by Race and Ethnicity **eTable 3.** Elixhauser Score by Race and Ethnicity and Acute Care Setting **eTable 4.** Racial and Ethnic Differences in Receiving Diagnostic Testing by Acute Care Setting: Full Regression Results **eTable 5.** Subanalysis of Association Between Race and Ethnicity and Diagnostic Testing by Zip Code Income Quartile, Emergency Department Discharges **eTable 6.** Subanalysis of Association Between Race and Ethnicity and Diagnostic Testing by Zip Code Income Quartile, Observation Stays **eTable 7.** Subanalysis of Association Between Race and Ethnicity and Diagnostic Testing by Zip Code Income Quartile, Inpatient Admissions **eTable 8.** Subanalysis of Association Between Race and Ethnicity and Diagnostic Testing for Medicaid Patients Under Age 65 **eTable 9.** Subanalysis of Association Between Race and Ethnicity and Diagnostic Testing for Self-Pay Patients Under Age 65 **eTable 10.** Subanalysis of Association Between Race and Ethnicity and Diagnostic Testing for Unhoused Patients in Maryland **eTable 11.** Subanalysis of Inpatient Hospitalizations That Did Not Begin in the ED **eTable 12.** Sensitivity Analysis—Racial and Ethnic Differences in Receiving Diagnostic Testing by Acute Care Setting, Adding Covariates for Sex, Age, and Elixhauser Score **eTable 13.** Sensitivity Analysis—Racial and Ethnic Differences in Receiving Diagnostic Testing by Acute Care Setting, Without Hospital-Specific Identifier Variable **eTable 14.** Sensitivity Analysis Using Visit Reason for Emergency Department and Observation Stays and Admission Diagnosis for Hospital Admissions **eTable 15.** Sensitivity Analysis of the Association Between Race and Diagnostic Testing Using Presentation Diagnosis Codes Rather Than Discharge Diagnosis Codes

Supplement 2.
Data Sharing Statement
