# Supplementary material for: Race and Ethnicity and Diagnostic Testing for Common Conditions in the Acute Care Setting
Source: JAMA Netw Open. 2024 Aug 27;7(8):e2430306. doi: 10.1001/jamanetworkopen.2024.30306 (PMC11350469; doi:10.1001/jamanetworkopen.2024.30306)
Supplement: Supplement 1. — eTable 1. Diagnostic and Procedural Codes for Metrics eTable 2. Payer Mix by Race and Ethnicity eTable 3. Elixhauser Score by Race and Ethnicity and Acute Care Setting eTable 4. Racial and Ethnic Differences in Receiving Diagnostic Testing by Acute Care Setting: Full Regression Results eTable 5. Subanalysis of Association Between Race and Ethnicity and Diagnostic Testing by Zip Code Income Quartile, Emergency Department Discharges eTable 6. Subanalysis of Association Between Race and Ethnicity and Diagnostic Testing by Zip Code Income Quartile, Observation Stays eTable 7. Subanalysis of Association Between Race and Ethnicity and Diagnostic Testing by Zip Code Income Quartile, Inpatient Admissions eTable 8. Subanalysis of Association Between Race and Ethnicity and Diagnostic Testing for Medicaid Patients Under Age 65 eTable 9. Subanalysis of Association Between Race and Ethnicity and Diagnostic Testing for Self-Pay Patients Under Age 65 eTable 10. Subanalysis of Association Between Race and Ethnicity and Diagnostic Testing for Unhoused Patients in Maryland eTable 11. Subanalysis of Inpatient Hospitalizations That Did Not Begin in the ED eTable 12. Sensitivity Analysis—Racial and Ethnic Differences in Receiving Diagnostic Testing by Acute Care Setting, Adding Covariates for Sex, Age, and Elixhauser Score eTable 13. Sensitivity Analysis—Racial and Ethnic Differences in Receiving Diagnostic Testing by Acute Care Setting, Without Hospital-Specific Identifier Variable eTable 14. Sensitivity Analysis Using Visit Reason for Emergency Department and Observation Stays and Admission Diagnosis for Hospital Admissions eTable 15. Sensitivity Analysis of the Association Between Race and Diagnostic Testing Using Presentation Diagnosis Codes Rather Than Discharge Diagnosis Codes [file jamanetwopen-e2430306-s001.pdf]

Supplemental Online Content

Ellenbogen MI, Weygandt PL, Newman-Toker D, Anderson A, Rim N, Brotman DJ. Race and ethnicity and diagnostic testing for common conditions in the acute care setting. *JAMA Netw Open*. 2024;7(8):e2430306. doi:10.1001/jamanetworkopen.2024.30306

eTable 1. Diagnostic and Procedural Codes for Metrics

eTable 2. Payer Mix by Race and Ethnicity

eTable 3. Elixhauser Score by Race and Ethnicity and Acute Care Setting

eTable 4. Racial and Ethnic Differences in Receiving Diagnostic Testing by Acute Care Setting: Full Regression Results

eTable 5. Subanalysis of Association Between Race and Ethnicity and Diagnostic Testing by Zip Code Income Quartile, Emergency Department Discharges

eTable 6. Subanalysis of Association Between Race and Ethnicity and Diagnostic Testing by Zip Code Income Quartile, Observation Stays

eTable 7. Subanalysis of Association Between Race and Ethnicity and Diagnostic Testing by Zip Code Income Quartile, Inpatient Admissions

eTable 8. Subanalysis of Association Between Race and Ethnicity and Diagnostic Testing for Medicaid Patients Under Age 65

eTable 9. Subanalysis of Association Between Race and Ethnicity and Diagnostic Testing for Self-Pay Patients Under Age 65

eTable 10. Subanalysis of Association Between Race and Ethnicity and Diagnostic Testing for Unhoused Patients in Maryland

eTable 11. Subanalysis of Inpatient Hospitalizations That Did Not Begin in the ED

eTable 12. Sensitivity Analysis—Racial and Ethnic Differences in Receiving Diagnostic Testing by Acute Care Setting, Adding Covariates for Sex, Age, and Elixhauser Score

eTable 13. Sensitivity Analysis—Racial and Ethnic Differences in Receiving Diagnostic Testing by Acute Care Setting, Without Hospital-Specific Identifier Variable

eTable 14. Sensitivity Analysis Using Visit Reason for Emergency Department and Observation Stays and Admission Diagnosis for Hospital Admissions

eTable 15. Sensitivity Analysis of the Association Between Race and Diagnostic Testing Using Presentation Diagnosis Codes Rather Than Discharge Diagnosis Codes

This supplemental material has been provided by the authors to give readers additional information about their work.

© 2024 Ellenbogen MI et al. *JAMA Network Open*.

Supplement Table 1 – Diagnostic and Procedural Codes for Metrics

Metric	*ICD10 Diagnosis Codes*	*ICD10 Procedure Codes*	*CPT Code*	*Revenue Code*
Nausea/vomiting and CT abdomen/pelvis or upper endoscopy	R110, R1110, R1111, R112	CT: BW2000Z BW200ZZ BW2010Z BW201ZZ BW20Y0Z BW20YZZ BW20ZZZ BW2100Z BW210ZZ BW2110Z BW211ZZ BW21Y0Z BW21YZZ BW21ZZZ BW2400Z BW240ZZ BW2410Z BW241ZZ BW24Y0Z BW24YZZ BW24ZZZ BW2500Z BW250ZZ BW2510Z BW251ZZ BW25Y0Z BW25YZZ BW25ZZZ<br><br>Upper endoscopy: 0DJ07ZZ 0DJ08ZZ 0DJ0XZZ 0DJ67ZZ 0DJ68ZZ 0DJ6XZZ	CT: 74176, 74177, 74178, 74170, 74160, 74150, 74174, 74175<br><br>Upper endoscopy: 43235, 43237, 43259	CT: 0350, 0352<br><br>Upper endoscopy: 0750 0759
Abdominal pain and CT abdomen/pelvis or upper endoscopy	R1010, R1011, R1012, R1013, R1030, R1031, R1032, R1033, R10811, R10812, R10813, R10814, R10815, R10816, R10817, R10819, R1083, R1084, R109	CT: BW2000Z BW200ZZ BW2010Z BW201ZZ BW20Y0Z BW20YZZ BW20ZZZ BW2100Z BW210ZZ BW2110Z BW211ZZ BW21Y0Z BW21YZZ BW21ZZZ BW2400Z BW240ZZ BW2410Z BW241ZZ BW24Y0Z BW24YZZ BW24ZZZ BW2500Z BW250ZZ BW2510Z BW251ZZ BW25Y0Z BW25YZZ BW25ZZZ BW2G00Z BW2G0ZZ BW2G10Z BW2G1ZZ BW2GY0Z BW2GYZZ BW2GZZZ<br><br>Upper endoscopy: 0DJ07ZZ 0DJ08ZZ 0DJ0XZZ 0DJ67ZZ 0DJ68ZZ 0DJ6XZZ	CT: 74176, 74177, 74178, 74170, 74160, 74150, 74174, 74175, 72194, 72193, 72192, 72191<br><br>Upper endoscopy: 43235, 43237, 43259	CT: 0350, 0352<br><br>Upper endoscopy: 0750 0759
Chest pain and CT chest or stress test	R071, R072, R0782, R0789, R079	CT: BB2400Z, BB240ZZ, BB2410Z, BB241ZZ, BB24Y0Z, BB24YZZ, BB24ZZZ, BW2400Z, BW240ZZ, BW2410Z, BW241ZZ, BW24Y0Z,	CT: 71260, 71250, 71270, 71275, 75574, 75572, 75571, 75573	CT: 0350, 0352

© 2024 Ellenbogen MI et al. *JAMA Network Open.*

		BW24YZZ, BW24ZZZ, BW2500Z, BW250ZZ, BW2510Z, BW251ZZ, BW25Y0Z, BW25YZZ, BW25ZZZ<br><br>Stress test: C2161ZZ, C216YZZ, C2261ZZ, C226YZZ, 4A12XM4	Stress test: 78451, 78452, 78453, 78454, 93015, 93016, 93017, 93018, 93350, 93351, 93352	Stress test: 0482
Syncope and CT brain or stress test*	R55	CT: B02000Z, B0200ZZ, B02010Z, B0201ZZ, B020Y0Z, B020YZZ, B020ZZZ, B32R0ZZ, B32R1ZZ, B32RYZZ, B32RZ2Z<br><br>Stress test: C2161ZZ, C216YZZ, C2261ZZ, C226YZZ, 4A12XM4	CT: 70460, 70450, 70470, 70496<br><br>Stress test: 78451, 78452, 78453, 78454, 93015, 93016, 93017, 93018, 93350, 93351, 93352	CT: 0350, 0351<br><br>Stress test: 0482

<u>Abbreviations:</u>
CPT – Current Procedural Terminology
CT – Computed Tomography
ICD – International Classification of Disease

*In the original paper of the derivation-validation process, this was treated as two separate metrics, but when applying this DII at the patient level, it is treated as a single measure.

© 2024 Ellenbogen MI et al. *JAMA Network Open.*

Supplement Table 2 – Payer Mix by Race

	Primary Payer						
**Race**	**Medicare**	**Medicaid**	**Commercial**	**Self-Pay**	**Other**	**Missing**	**Total**
Asian	8,045 (0.9)	7,699 (0.9)	22,683 (1.7)	4,341 (0.9)	2,601 (2.1)	45 (0.9)	45,414 (1.2)
Black	206,899 (24.2)	310,524 (36.0)	344,389 (25.2)	164,317 (35.4)	27,402 (21.8)	2,044 (40.4)	1,055,575 (28.7)
Hispanic	33,804 (4.0)	71,396 (8.3)	93,225 (6.8)	75,221 (16.2)	26,526 (21.1)	161 (3.2)	300,333 (8.2)
White	586,050 (68.5)	442,144 (51.2)	856,368 (62.5)	193,107 (41.6)	60,166 (47.9)	2,500 (49.4)	2,140,335 (58.1)
Other	16,845 (2.0)	28,353 (3.23)	42,296 (3.1)	22,115 (4.8)	7,881 (6.3)	283 (5.6)	117,773 (3.2)
Missing	3,403 (0.40)	3,386 (0.4)	10,641 (0.8)	5,195 (1.1)	968 (0.8)	32 (0.6)	23,625 (0.6)
Total	855,046 (100.0)	863,502 (100.0)	1,369,602 (100.0)	464,296 (100.0)	125,544 (100.0)	5,065 (100.0)	3,683,055 (100)

Other is defined as mixed-race, Native American, and patients who selected other as their race.

© 2024 Ellenbogen MI et al. *JAMA Network Open*.

Supplement Table 3 – Elixhauser Score by Race and Acute Care Setting

	**Emergency Department**	**Observation Stay**	**Inpatient**
Total Visits - Number (%)	2,969,974 (80.6)	618,973 (16.8)	94,108 (2.6)
**Race**<br>Elixhauser score (SD)			
Asian	0.6 (0.9)	1.9 (1.5)	2.9 (1.9)
Black	0.9 (1.2)	2.6 (1.8)	3.7 (2.0)
Hispanic	0.6 (0.9)	1.8 (1.6)	2.8 (1.9)
White	0.9 (1.2)	2.5 (1.8)	3.5 (2.0)
Other	0.6 (0.9)	2.1 (1.6)	3.0 (1.9)
Missing	0.6 (0.9)	1.9 (1.6)	2.8 (1.8)
Entire Cohort	0.8 (1.1)	2.5 (1.7)	3.5 (2.0)

SD – Standard Deviation

Other is defined as mixed-race, Native American, and patients who selected other as their race.

© 2024 Ellenbogen MI et al. *JAMA Network Open.*

Supplement Table 4 – Racial Differences in Receiving Diagnostic Testing by Acute Care Setting, Full Regression Results

Setting	Variable	Odds Ratio	95% CI	p-value
ED	RACE			
	Asian (ref: White)	0.91	0.89-0.94	<0.001
	Black	0.74	0.72-0.75	<0.001
	Hispanic	1.00	0.98-1.02	0.93
	Other	0.93	0.89-0.97	0.002
	Missing	0.81	0.76-0.85	<0.001
	PAYER			
	Medicaid (ref: Medicare)	0.71	0.70-0.73	<0.001
	Commercial	0.86	0.84-0.88	<0.001
	Self-pay	0.66	0.65-0.68	<0.001
	Other	0.83	0.81-0.86	<0.001
	Missing	0.93	0.87-1.00	0.04
	ZIP CODE INCOME QUARTILE			
	Q2 (ref: lowest income quartile)	1.02	1.01-1.04	0.002
	Q3	1.04	1.02-1.06	<0.001
	Q4	1.04	1.01-1.06	0.02
Observation	RACE			
	Asian	1.08	1.03-1.13	0.002
	Black	0.92	0.90-0.94	<0.001
	Hispanic	1.03	1.00-1.07	0.06
	Other	1.02	0.99-1.06	0.22
	Missing	1.11	1.03-1.19	0.009
	PAYER			
	Medicaid	1.01	0.98-1.03	0.64
	Commercial	1.27	1.22-1.33	<0.001
	Self-pay	1.21	1.14-1.28	<0.001
	Other	1.26	1.20-1.31	<0.001
	Missing	1.44	1.23-1.68	<0.001
	ZIP CODE INCOME QUARTILE			
	Q2	1.05	1.02-1.07	<0.001
	Q3	1.06	1.03-1.09	<0.001
	Q4	1.05	1.01-1.09	0.02
Inpatient	RACE			
	Asian	1.13	1.01-1.27	0.04
	Black	1.00	0.95-1.05	0.90
	Hispanic	1.09	1.02-1.17	0.02
	Other	1.01	0.92-1.10	0.85
	Missing	0.83	0.67-1.03	0.09

© 2024 Ellenbogen MI et al. *JAMA Network Open.*

	PAYER			
	Medicaid	0.91	0.85-0.96	0.001
	Commercial	0.99	0.94-1.04	0.62
	Self-pay	0.99	0.92-1.08	0.86
	Other	0.96	0.86-1.07	0.43
	Missing	1.42	0.80-2.53	0.24
	ZIP CODE INCOME QUARTILE			
	Q2	1.08	1.01-1.15	0.02
	Q3	1.13	1.05-1.21	0.002
	Q4	1.11	1.03-1.19	0.007

CI – Confidence Interval
ED – Emergency Department
Q – Quartile

Generalized linear model with a hospital-specific indicator variable, adjusting for primary payer and ZIP code income quartile

Other is defined as mixed-race, Native American, and patients who selected other as their race.

© 2024 Ellenbogen MI et al. *JAMA Network Open*.

Supplement Table 5 – Sub-Analysis of Relationship Between Race and Diagnostic Testing by ZIP Code Income Quartile, *Emergency Department Discharges*

ZIP Code Income Quartile	Variable	Odds Ratio	95%	CI	p-value
Q1 (lowest income)	RACE				
	Asian (ref: White)	1.03	0.97	1.09	0.40
	Black	0.73	0.71	0.74	<0.001
	Hispanic	1.02	0.99	1.05	0.13
	Other	0.94	0.91	0.97	<0.001
	Missing	0.78	0.71	0.86	<0.001
	PAYER				
	Medicaid (ref: Medicare)	0.73	0.71	0.76	<0.001
	Commercial	0.89	0.87	0.92	<0.001
	Self-pay	0.68	0.66	0.71	<0.001
	Other	0.88	0.84	0.91	<0.001
	Missing	0.98	0.89	1.09	0.73
Q2	RACE				
	Asian	0.93	0.86	1.01	0.08
	Black	0.72	0.71	0.74	<0.001
	Hispanic	1.00	0.96	1.04	0.91
	Other	0.96	0.84	1.10	0.57
	Missing	0.83	0.76	0.90	<0.001
	PAYER				
	Medicaid	0.70	0.68	0.73	<0.001
	Commercial	0.85	0.83	0.88	<0.001
	Self-pay	0.65	0.62	0.68	<0.001
	Other	0.80	0.77	0.84	<0.001
	Missing	0.92	0.82	1.04	0.18
Q3	RACE				
	Asian	0.89	0.84	0.95	<0.001
	Black	0.75	0.73	0.78	<0.001
	Hispanic	1.00	0.97	1.03	0.99
	Other	0.91	0.87	0.94	<0.001
	Missing	0.82	0.75	0.88	<0.001
	PAYER				
	Medicaid	0.69	0.66	0.72	<0.001
	Commercial	0.84	0.81	0.86	<0.001
	Self-pay	0.65	0.63	0.68	<0.001
	Other	0.82	0.78	0.86	<0.001

© 2024 Ellenbogen MI et al. *JAMA Network Open.*

	Missing	0.87	0.77	0.98	0.02
Q4	RACE				
	Asian	0.90	0.86	0.93	<0.001
	Black	0.79	0.78	0.81	<0.001
	Hispanic	1.00	0.97	1.04	0.92
	Other	0.90	0.87	0.94	<0.001
	Missing	0.81	0.73	0.90	<0.001
	PAYER				
	Medicaid	0.72	0.69	0.75	<0.001
	Commercial	0.83	0.81	0.86	<0.001
	Self-pay	0.66	0.63	0.69	<0.001
	Other	0.81	0.77	0.85	<0.001
	Missing	0.95	0.88	1.04	0.26

CI – Confidence Interval
Q – Quartile

Sub-analysis by ZIP code income quartile of ED discharges using a generalized linear model with a hospital-specific indicator variable, adjusting for primary payer

Other is defined as mixed-race, Native American, and patients who selected other as their race.

© 2024 Ellenbogen MI et al. *JAMA Network Open*.

Supplement Table 6 – Sub-Analysis of Relationship Between Race and Diagnostic Testing by ZIP Code Income Quartile, *Observation Stays*

**ZIP Code Income Quartile**	**Variable**	**Odds Ratio**	**95%**	**CI**	**p-value**
Q1 (lowest income)	RACE				
	Asian (ref: White)	1.25	1.09	1.45	0.002
	Black	0.92	0.90	0.96	<0.001
	Hispanic	1.02	0.96	1.08	0.54
	Other	0.98	0.91	1.07	0.70
	Missing	1.07	0.94	1.21	0.32
	PAYER				
	Medicaid (ref: Medicare)	0.98	0.95	1.02	0.39
	Commercial	1.31	1.24	1.40	<0.001
	Self-pay	1.23	1.13	1.34	<0.001
	Other	1.21	1.12	1.29	<0.001
	Missing	1.42	1.22	1.66	<0.001
Q2	RACE				
	Asian	1.15	1.01	1.30	0.03
	Black	0.93	0.88	0.97	0.003
	Hispanic	1.08	1.00	1.17	0.060
	Other	1.17	1.07	1.27	<0.001
	Missing	1.12	0.91	1.38	0.30
	PAYER				
	Medicaid	1.00	0.95	1.04	0.85
	Commercial	1.34	1.24	1.45	<0.001
	Self-pay	1.20	1.10	1.30	<0.001
	Other	1.31	1.21	1.42	<0.001
	Missing	1.47	1.15	1.89	0.002
Q3	RACE				
	Asian	1.05	0.96	1.15	0.25
	Black	0.92	0.88	0.95	<0.001
	Hispanic	1.04	0.99	1.10	0.13
	Other	1.02	0.94	1.11	0.61
	Missing	1.17	1.03	1.34	0.02
	PAYER				
	Medicaid	1.05	1.00	1.10	0.04
	Commercial	1.27	1.20	1.34	<0.001

© 2024 Ellenbogen MI et al. *JAMA Network Open.*

	Self-pay	1.21	1.13	1.30	<0.001
	Other	1.20	1.09	1.32	<0.001
	Missing	1.32	1.00	1.74	0.05
Q4	RACE				
	Asian	1.04	0.98	1.11	0.19
	Black	0.93	0.90	0.96	<0.001
	Hispanic	1.03	0.97	1.09	0.29
	Other	0.98	0.92	1.04	0.44
	Missing	1.09	0.96	1.24	0.21
	PAYER				
	Medicaid	1.06	1.00	1.11	0.05
	Commercial	1.19	1.13	1.26	<0.001
	Self-pay	1.21	1.09	1.34	0.001
	Other	1.30	1.20	1.40	<0.001
	Missing	1.84	1.25	2.71	0.002

CI – Confidence Interval
Q – Quartile

Sub-analysis by ZIP code income quartile of observation stays using a generalized linear model with a hospital-specific indicator variable, adjusting for primary payer

Other is defined as mixed-race, Native American, and patients who selected other as their race.

© 2024 Ellenbogen MI et al. *JAMA Network Open*.

Supplement Table 7 – Sub-Analysis of Relationship Between Race and Diagnostic Testing by ZIP Code Income Quartile, *Inpatient Admissions*

**ZIP Code Income Quartile**	**Variable**	**Odds Ratio**	**95%**	**CI**	**p-value**
Q1 (lowest income)	RACE				
	Asian (ref: White)	1.42	0.95	2.13	0.09
	Black	1.07	0.99	1.16	0.09
	Hispanic	1.13	0.98	1.31	0.10
	Other	1.08	0.93	1.25	0.33
	Missing	0.92	0.65	1.31	0.64
	PAYER				
	Medicaid (ref: Medicare)	0.94	0.87	1.01	0.08
	Commercial	1.07	1.01	1.14	0.02
	Self-pay	1.01	0.91	1.12	0.90
	Other	1.11	0.95	1.30	0.19
	Missing	1.13	0.36	3.54	0.84
Q2	RACE				
	Asian	1.14	0.83	1.58	0.42
	Black	0.97	0.90	1.05	0.52
	Hispanic	1.12	0.94	1.34	0.20
	Other	1.07	0.91	1.25	0.40
	Missing	0.89	0.51	1.55	0.67
	PAYER				
	Medicaid	0.90	0.81	1.00	0.05
	Commercial	1.02	0.92	1.12	0.74
	Self-pay	1.02	0.85	1.22	0.84
	Other	0.80	0.65	1.00	0.05
	Missing	1.75	0.85	3.59	0.13
Q3	RACE				
	Asian	1.06	0.80	1.41	0.69
	Black	0.89	0.81	0.98	0.02
	Hispanic	0.98	0.84	1.14	0.77
	Other	0.91	0.74	1.13	0.40
	Missing	0.68	0.42	1.10	0.12
	PAYER				
	Medicaid	0.94	0.83	1.06	0.32

© 2024 Ellenbogen MI et al. *JAMA Network Open*.

	Commercial	0.99	0.90	1.09	0.79
	Self-pay	1.02	0.81	1.28	0.89
	Other	0.98	0.80	1.20	0.85
	Missing	1.67	0.58	4.80	0.34
Q4	RACE				
	Asian	1.11	0.96	1.29	0.16
	Black	0.97	0.89	1.05	0.45
	Hispanic	1.14	0.96	1.35	0.14
	Other	0.96	0.83	1.13	0.66
	Missing	0.88	0.65	1.18	0.40
	PAYER				
	Medicaid	0.84	0.75	0.94	0.002
	Commercial	0.89	0.82	0.97	0.009
	Self-pay	1.00	0.85	1.19	0.97
	Other	0.97	0.81	1.17	0.77
	Missing	0.51	0.07	3.64	0.51

CI – Confidence Interval
Q – Quartile

Sub-analysis by ZIP code income quartile of inpatient hospitalizations using a generalized linear model with a hospital-specific indicator variable, adjusting for primary payer

Other is defined as mixed-race, Native American, and patients who selected other as their race.

© 2024 Ellenbogen MI et al. *JAMA Network Open*.

Supplement Table 8 – Sub-Analysis of Relationship Between Race and Diagnostic Testing for Medicaid Patients Under Age 65

Setting	Variable	Odds Ratio	95%	CI	p-value
ED	RACE				
	Asian (ref: White)	0.99	0.93	1.05	0.73
	Black	0.72	0.70	0.73	<0.001
	Hispanic	0.93	0.90	0.96	<0.001
	Other	0.92	0.88	0.96	<0.001
	Missing	0.80	0.72	0.90	<0.001
	ZIP CODE INCOME QUARTILE				
	Q2 (ref: lowest income quartile)	1.03	1.00	1.05	0.02
	Q3	1.04	1.01	1.07	0.003
	Q4	1.08	1.04	1.11	<0.001
Observation	RACE				
	Asian	1.17	1.03	1.33	0.01
	Black	0.94	0.90	0.98	0.002
	Hispanic	1.08	1.01	1.15	0.03
	Other	1.03	0.95	1.13	0.48
	Missing	1.25	1.02	1.54	0.04
	ZIP CODE INCOME QUARTILE				
	Q2	1.07	1.01	1.13	0.02
	Q3	1.09	1.04	1.15	<0.001
	Q4	1.10	1.02	1.19	0.008
Inpatient	RACE				
	Asian	1.51	1.03	2.22	0.04
	Black	1.01	0.91	1.11	0.88
	Hispanic	1.09	0.92	1.30	0.32
	Other	1.35	1.06	1.70	0.01
	Missing	0.85	0.50	1.43	0.54
	ZIP CODE INCOME QUARTILE				
	Q2	1.01	0.91	1.14	0.80
	Q3	1.04	0.90	1.20	0.60
	Q4	0.93	0.80	1.09	0.36

CI – Confidence Interval
Q – Quartile

Sub-analysis of Medicaid patients under age 65 using a generalized linear model with a hospital-specific indicator variable, adjusting for ZIP code income quartile

Other is defined as mixed-race, Native American, and patients who selected other as their race.

© 2024 Ellenbogen MI et al. *JAMA Network Open.*

Supplement Table 9 – Sub-Analysis of Relationship Between Race and Diagnostic Testing for Self-Pay Patients Under Age 65

Setting	Variable	Odds Ratio	95%	CI	p-value
ED	RACE				
	Asian (ref: White)	0.99	0.92	1.07	0.83
	Black	0.65	0.63	0.66	<0.001
	Hispanic	1.05	1.02	1.09	0.002
	Other	0.94	0.87	1.01	0.08
	Missing	0.61	0.54	0.70	<0.001
	ZIP CODE INCOME QUARTILE				
	Q2 (ref: lowest income quartile)	1.00	0.97	1.02	0.79
	Q3	1.01	0.98	1.04	0.37
	Q4	1.00	0.96	1.04	0.92
Observation	RACE				
	Asian	1.15	0.93	1.42	0.19
	Black	0.93	0.88	0.99	0.03
	Hispanic	1.11	1.02	1.20	0.02
	Other	1.03	0.93	1.15	0.54
	Missing	1.02	0.79	1.32	0.86
	ZIP CODE INCOME QUARTILE				
	Q2	0.97	0.91	1.04	0.38
	Q3	1.04	0.97	1.12	0.28
	Q4	1.05	0.95	1.16	0.36
Inpatient	RACE				
	Asian	0.83	0.41	1.70	0.61
	Black	1.01	0.83	1.23	0.91
	Hispanic	1.29	1.00	1.66	0.05
	Other	0.98	0.71	1.35	0.91
	Missing	0.53	0.27	1.06	0.07
	ZIP CODE INCOME QUARTILE				
	Q2	1.19	0.94	1.49	0.14
	Q3	1.23	0.95	1.59	0.12
	Q4	1.35	1.01	1.81	0.05

CI – Confidence Interval
ED – Emergency Department
Q – Quartile

Sub-analysis of self-pay patients using a generalized linear model with a hospital-specific indicator variable, adjusting for ZIP code income quartile

© 2024 Ellenbogen MI et al. *JAMA Network Open.*

Other is defined as mixed-race, Native American, and patients who selected other as their race.

© 2024 Ellenbogen MI et al. *JAMA Network Open*.

Supplement Table 10 – Sub-Analysis of Relationship Between Race and Diagnostic Testing for Homeless Patients in Maryland

Setting	Race	Odds Ratio	95%	CI	p-value
ED	Asian (ref: White)	1.86	0.23	15.08	0.56
	Black	0.69	0.48	1.01	0.06
	Hispanic	0.73	0.30	1.78	0.49
	Other	0.77	0.34	1.74	0.53
	Missing	0.64	0.18	2.30	0.49
Observation	Asian	*			
	Black	1.92	0.97	3.78	0.06
	Hispanic	1.86	0.57	6.02	0.30
	Other	0.59	0.19	1.82	0.36
	Missing	*			
Inpatient	Asian	*			
	Black	1.53	0.08	29.59	0.78
	Hispanic	0.34	0.03	4.25	0.40
	Other	8.17	0.48	140.01	0.15
	Missing	*			

CI – Confidence Interval
ED – Emergency Department

* - Too few observations to calculate odds ratios

Sub-analysis of homeless patients (based on HCUP indicator variable for homelessness) using a generalized linear model with a hospital-specific indicator variable; including only Maryland as the other states do not provide data on homelessness.

Maryland had 2,377 homeless patient encounters out of a total of 684,127 (0.35%).

Other is defined as mixed-race, Native American, and patients who selected other as their race.

© 2024 Ellenbogen MI et al. *JAMA Network Open*.

Supplement Table 11 – Sub-Analysis of Inpatient Hospitalizations That Did Not Begin in the ED

Variable	Odds Ratio	95% CI	p-value
RACE			
Asian (ref: White)	1.21	0.76-1.92	0.42
Black	1.05	0.94-1.18	0.38
Hispanic	1.35	1.07-1.71	0.01
Other	1.30	0.94-1.79	0.12
Missing	0.68	0.45-1.02	0.06
PAYER			
Medicaid (ref: Medicare)	0.88	0.78-0.99	0.03
Commercial	0.99	0.90-1.09	0.82
Self-pay	0.87	0.68-1.12	0.28
Other	0.89	0.65-1.20	0.44
Missing	4.37	1.25-15.22	0.02
ZIP CODE INCOME QUARTILE			
Q2 (ref: lowest income quartile)	1.10	0.98-1.23	0.09
Q3	1.16	0.98-1.36	0.08
Q4	1.05	0.87-1.27	0.62

CI – Confidence Interval
ED – Emergency Department
Q – Quartile

Generalized linear model with a hospital-specific indicator variable, adjusting for primary payer and ZIP code income quartile

There were 94,108 inpatient encounters, of which 82,204 (87.3%) began in the ED. This model only includes the 12.6% (11,904 inpatient encounters) that did not begin in the ED.

Other is defined as mixed-race, Native American, and patients who selected other as their race.

© 2024 Ellenbogen MI et al. *JAMA Network Open*.

Supplement Table 12 – Sensitivity Analysis – Racial Differences in Receiving Diagnostic Testing by Acute Care Setting, Adding Covariates for Gender, Age, and Elixhauser Score

Setting	Variable	Odds Ratio	95% CI	p-value
ED	RACE			
	Asian (ref: White)	0.91	0.88-0.94	<0.001
	Black	0.75	0.74-0.77	<0.001
	Hispanic	1.03	1.01-1.05	0.01
	Other	0.96	0.92-1.00	0.06
	Missing	0.83	0.78-0.87	<0.001
	PAYER			
	Medicaid (ref: Medicare)	1.01	0.99-1.02	0.57
	Commercial	1.14	1.12-1.16	<0.001
	Self-pay	0.97	0.96-0.99	0.006
	Other	1.14	1.11-1.16	<0.001
	Missing	1.21	1.16-1.26	<0.001
	ZIP CODE INCOME QUARTILE			
	Q2 (ref: lowest income quartile)	1.03	1.01-1.04	0.001
	Q3	1.04	1.01-1.06	0.002
	Q4	1.03	1.00-1.06	0.08
	FEMALE	1.15	1.14-1.17	<0.001
	AGE	1.012	1.011-1.013	<0.001
	ELIXHASUER COMORBIDITY INDEX	1.02	1.01-1.03	<0.001
Observation	RACE			
	Asian	1.06	1.01-1.11	0.02
	Black	0.94	0.92-0.96	<0.001
	Hispanic	1.03	0.99-1.06	0.11
	Other	1.02	0.98-1.06	0.42
	Missing	1.09	1.02-1.18	0.02
	PAYER			
	Medicaid	1.03	0.99-1.07	0.10
	Commercial	1.25	1.20-1.29	<0.001
	Self-pay	1.19	1.14-1.25	<0.001
	Other	1.26	1.20-1.31	<0.001
	Missing	1.40	1.20-1.64	<0.001
	ZIP CODE INCOME QUARTILE			
	Q2	1.04	1.02-1.07	0.001
	Q3	1.05	1.02-1.07	0.001
	Q4	1.02	0.99-1.06	0.18
	FEMALE	1.12	1.10-1.14	<0.001
	AGE	1.003	1.002-1.005	<0.001

© 2024 Ellenbogen MI et al. *JAMA Network Open*.

	ELIXHASUER COMORBIDITY INDEX	0.92	0.91-0.93	<0.001
Inpatient	RACE			
	Asian	1.12	1.00-1.25	0.06
	Black	1.03	0.98-1.08	0.28
	Hispanic	1.11	1.03-1.19	0.006
	Other	1.02	0.93-1.11	0.71
	Missing	0.83	0.67-1.04	0.10
	PAYER			
	Medicaid	1.02	0.96-1.08	0.49
	Commercial	1.06	1.02-1.11	0.009
	Self-pay	1.11	1.02-1.20	0.02
	Other	1.04	0.94-1.16	0.43
	Missing	1.50	0.84-2.69	0.17
	ZIP CODE INCOME QUARTILE			
	Q2	1.07	1.01-1.14	0.02
	Q3	1.11	1.04-1.20	0.004
	Q4	1.09	1.01-1.17	0.02
	FEMALE	1.09	1.06-1.13	<0.001
	AGE	1.01	1.00-1.01	<0.001
	ELIXHASUER COMORBIDITY INDEX	0.97	0.96-0.98	<0.001

CI – Confidence Interval
ED – Emergency Department
Q – Quartile

Generalized linear model with a hospital-specific indicator variable, adjusting for primary payer, ZIP code income quartile, gender, age (continuous), and Elixhauser comorbidity index (continuous)

Other is defined as mixed-race, Native American, and patients who selected other as their race.

© 2024 Ellenbogen MI et al. *JAMA Network Open.*

Supplement Table 13 – Sensitivity Analysis – Racial Differences in Receiving Diagnostic Testing by Acute Care Setting, Without Hospital-Specific Identifier Variable

Setting	Variable	Odds Ratio	95% CI	p-value
ED	RACE			
	Asian (ref: White)	0.85	0.83-0.87	<0.001
	Black	0.68	0.68-0.69	<0.001
	Hispanic	0.86	0.85-0.87	<0.001
	Other	0.87	0.86-0.88	<0.001
	Missing	0.73	0.71-0.75	<0.001
	PAYER			
	Medicaid (ref: Medicare)	0.71	0.70-0.71	<0.001
	Commercial	0.87	0.86-0.87	<0.001
	Self-pay	0.66	0.66-0.67	<0.001
	Other	0.82	0.80-0.83	<0.001
	Missing	1.03	0.96-1.09	0.44
	ZIP CODE INCOME QUARTILE			
	Q2 (ref: lowest income quartile)	1.10	1.10-1.11	<0.001
	Q3	1.14	1.13-1.15	<0.001
	Q4	1.17	1.16-1.18	<0.001
Observation	RACE			
	Asian	0.95	0.91-0.99	0.02
	Black	0.89	0.88-0.91	<0.001
	Hispanic	0.90	0.88-0.92	<0.001
	Other	0.84	0.82-0.87	<0.001
	Missing	1.04	0.97-1.12	0.23
	PAYER			
	Medicaid	0.97	0.95-0.98	<0.001
	Commercial	1.27	1.26-1.29	<0.001
	Self-pay	1.21	1.18-1.24	<0.001
	Other	1.29	1.25-1.33	<0.001
	Missing	1.82	1.54-2.16	<0.001
	ZIP CODE INCOME QUARTILE			
	Q2	1.15	1.13-1.16	<0.001
	Q3	1.05	1.03-1.07	<0.001
	Q4	0.98	0.97-1.00	0.009
Inpatient	RACE			
	Asian	1.02	0.91-1.15	0.70
	Black	1.00	0.97-1.03	0.87
	Hispanic	1.08	1.02-1.14	0.009
	Other	1.10	1.01-1.20	0.02

© 2024 Ellenbogen MI et al. *JAMA Network Open*.

	Missing	0.82	0.69-0.98	0.03
	PAYER			
	Medicaid	0.90	0.87-0.94	<0.001
	Commercial	0.96	0.93-0.99	0.01
	Self-pay	0.96	0.89-1.02	0.20
	Other	0.95	0.87-1.03	0.20
	Missing	1.27	0.76-2.13	0.37
	ZIP CODE INCOME QUARTILE			
	Q2	1.09	1.05-1.13	<0.001
	Q3	1.18	1.14-1.23	<0.001
	Q4	1.13	1.09-1.17	<0.001

CI – Confidence Interval
ED – Emergency Department
Q – Quartile

Generalized linear model, adjusting for primary payer and ZIP code income quartile

Other is defined as mixed-race, Native American, and patients who selected other as their race.

© 2024 Ellenbogen MI et al. *JAMA Network Open*.

Supplement Table 14 – Sensitivity Analysis Using Visit Reason for Emergency Department and Observation Stays and Admission Diagnosis for Hospital Admissions

	Presentation Diagnoses				
**Race**	**Nausea/Vomiting**	**Abdominal Pain**	**Chest Pain**	**Syncope**	**Total**
Asian	7,920 (1.1)	35,337 (1.3)	24,923 (1.4)	6,228 (1.7)	74,408 (1.4)
Black	194,310 (27.9)	678,116 (25.8)	515,740 (28.2)	83,902 (22.6)	1,472,068 (26.6)
Hispanic	57,881 (8.3)	304,720 (11.6)	142,580 (7.8)	21,343 (5.7)	526,524 (9.5)
White	410,798 (59.0)	1,499,012 (57.0)	1,076,620 (58.9)	246,203 (66.3)	3,232,633 (58.5)
Other	21,293 (3.1)	97,478 (3.7)	56,762 (3.1)	11,198 (3.0)	186,731 (3.4)
Missing	4,049 (0.6)	17,611 (0.7)	11,378 (0.6)	2,773 (0.8)	35,811 (0.7)
Total	696,251 (100.0)	2,632,274 (100.0)	1,828,003 (100.0)	371,647 (100.0)	5,528,175 (100.0)

Other is defined as mixed-race, Native American, and patients who selected other as their race.

© 2024 Ellenbogen MI et al. *JAMA Network Open.*

Supplement Table 15 – Sensitivity Analysis of the Relationship Between Race and Diagnostic Testing Using Presentation Diagnosis Codes Rather Than Discharge Diagnosis Codes

Setting	Variable	Odds Ratio	95%	CI	p-value
ED	RACE				
	Asian (ref: White)	0.91	0.88	0.94	<0.001
	Black	0.65	0.64	0.66	<0.001
	Hispanic	0.90	0.88	0.92	<0.001
	Other	0.87	0.85	0.89	<0.001
	Missing	0.79	0.75	0.84	<0.001
	PAYER				
	Medicaid (ref: Medicare)	0.64	0.62	0.66	<0.001
	Commercial	0.91	0.89	0.93	<0.001
	Self-pay	0.66	0.65	0.68	<0.001
	Other	0.81	0.78	0.83	<0.001
	Missing	0.88	0.76	1.02	0.10
	ZIP CODE INCOME QUARTILE				
	Q2 (ref: lowest income quartile)	1.04	1.02	1.06	<0.001
	Q3	1.07	1.05	1.09	<0.001
	Q4	1.10	1.07	1.13	<0.001
Observation	RACE				
	Asian	1.05	1.00	1.10	0.05
	Black	0.90	0.87	0.92	<0.001
	Hispanic	0.98	0.94	1.03	0.49
	Other	1.04	1.00	1.07	0.05
	Missing	1.06	0.99	1.13	0.09
	PAYER				
	Medicaid	0.99	0.96	1.02	0.44
	Commercial	1.23	1.18	1.28	<0.001
	Self-pay	1.14	1.07	1.22	<0.001
	Other	1.21	1.14	1.29	<0.001
	Missing	1.39	1.16	1.65	<0.001
	ZIP CODE INCOME QUARTILE				
	Q2	1.04	1.02	1.07	0.002
	Q3	1.06	1.02	1.10	0.002
	Q4	1.06	1.01	1.12	0.01
Inpatient	RACE				
	Asian	1.05	0.97	1.13	0.24

© 2024 Ellenbogen MI et al. *JAMA Network Open*.

	Black	1.03	0.99	1.07	0.13
	Hispanic	1.10	1.05	1.15	<0.001
	Other	1.00	0.95	1.06	0.92
	Missing	0.79	0.72	0.88	<0.001
	PAYER				
	Medicaid	0.91	0.88	0.94	<0.001
	Commercial	0.98	0.95	1.01	0.17
	Self-pay	0.95	0.92	0.98	0.004
	Other	0.94	0.89	1.00	0.06
	Missing	0.98	0.81	1.18	0.80
	ZIP CODE INCOME QUARTILE				
	Q2	1.07	1.01	1.13	0.02
	Q3	1.12	1.06	1.18	<0.001
	Q4	1.11	1.05	1.18	<0.001

CI – Confidence Interval
ED – Emergency Department
Q – Quartile

Generalized linear model with a hospital-specific indicator variable, adjusting for primary payer and ZIP code income quartile

Other is defined as mixed-race, Native American, and patients who selected other as their race.

© 2024 Ellenbogen MI et al. *JAMA Network Open*.
