# Supplementary material for: Race and Ethnicity and Diagnostic Testing for Common Conditions in the Acute Care Setting
Source: JAMA Netw Open. 2024 Aug 27;7(8):e2430306. doi: 10.1001/jamanetworkopen.2024.30306 (PMC11350469; doi:10.1001/jamanetworkopen.2024.30306)
Supplement: Supplement 2. — Data Sharing Statement [file jamanetwopen-e2430306-s002.pdf]

Data Sharing Statement

Ellenbogen. Race and Ethnicity and Diagnostic Testing for Common Conditions in the Acute Care Setting. *JAMA Netw Open*. Published online August 27, 2024. doi:10.1001/jamanetworkopen.2024.30306

Data
Data available: No

Additional Information
Explanation for why data not available: We cannot share the data due to an HCUP DUA. However, we are happy to share the code.
